# Steroid treatment response to post SARS-CoV-2 PANS symptoms: Case series

**DOI:** 10.3389/fneur.2023.1085948

**Published:** 2023-02-14

**Authors:** Stefano Berloffa, Andrea Salvati, Gloria Pantalone, Ludovica Falcioni, Micaela M. Rizzi, Francesca Naldini, Gabriele Masi, Antonella Gagliano

**Affiliations:** ^1^IRCCS Stella Maris, Scientific Institute of Child Neurology and Psychiatry, Pisa, Italy; ^2^Department of Clinical and Experimental Medicine, University of Pisa, Pisa, Italy; ^3^Child and Adolescent Neuropsychiatry Unit, Department of Biomedical Science, University of Cagliari & “A. Cao” Pediatric Hospital, Brotzu Hospital Trust, Cagliari, Italy; ^4^Neurology Unit, Department of Neurology of Health Science, Magna Graecia University of Catanzaro, Catanzaro, Italy

**Keywords:** SARS-CoV-2, TIC, pediatric acute-onset neuropsychiatric syndrome (PANS), steroid, neuropsychiatric disorder

## Abstract

**Background:**

Pediatric acute-onset neuropsychiatric syndrome (PANS) is characterized by a wide spectrum of symptoms, including the onset of obsessive-compulsive disorder and/or severely restricted food intake, associated with emotional symptoms, behavioral symptoms, developmental regression, and somatic symptoms. Among the possible triggering agents, infectious agents have been extensively explored. More recently, sporadic case reports describe a possible association between PANS and SARS-CoV-2 infection but data on clinical presentation and treatment are still scarce.

**Methods:**

We describe a case series (10 children) with acute onset or relapse of PANS symptoms after SARS-CoV-2 infection. Standardized measures (CBCL, CPRS, C-GAS, CGI-S, Y-BOCS, PANSS, and YGTSS) were used to describe the clinical picture. The efficacy of a pulse treatment with steroids for three consecutive months was assessed.

**Results:**

Our data suggest that the clinical presentation of the COVID-19-triggered PANS is largely similar to that reported in typical PANS, including acute onset, with OCD and/or eating disorders, and associated symptoms. Our data suggest that treatment with corticosteroids may be beneficial for both global clinical severity and global functioning. No serious adverse effects were observed. Both OCD symptoms and tics consistently improved. Among psychiatric symptoms, affective and oppositional symptoms appeared more sensitive to the steroid treatment than the other symptoms.

**Conclusion:**

Our study confirms that COVID-19 infection in children and adolescents could trigger acute-onset neuropsychiatric symptoms. Thus, in children and adolescents with COVID-19, a specific neuropsychiatric follow-up should be routinely included. Even if a small sample size and a follow-up with only two points (baseline and endpoint, after 8 weeks) limit the conclusions, it seems that steroid treatment in the acute phase may be beneficial and well tolerated.

## Introduction

Pediatric acute-onset neuropsychiatric syndrome (PANS) is a clinically defined disorder with both physical and psychiatric presentations, acute or sub-acute onset, and a possible waxing and waning course, usually triggered by autoimmune/inflammatory mechanisms. The clinical presentation may be heterogeneous, with abrupt-onset obsessive-compulsive disorder and/or severely restricted food intake, associated with emotional symptoms (anxiety, depression, irritability, or mood lability), behavioral symptoms (impulsive, defiant, or aggressive behavior), developmental regression (deterioration of school performances, cognitive and attentional deficits, and ADHD-like symptoms), and somatic symptoms (sleep disorders, enuresis or urinary frequency, and motor or sensory abnormalities), not better explained by concurrent medical disorders (1).

The pathogenesis of PANS comprises multiple and reciprocally interacting mechanisms, including endocrine/metabolic, post-infectious, and autoimmune reactivity. These factors interfere with the neurochemical system balance of the brain, generating a neuroinflammatory condition (2). It is assumed that the central nervous system (CNS) is first affected by an immune reaction triggered by infections or other environmental factors and that the persistence of the immunological imbalance, even after the resolution of the acute phase has passed, maintains the inflammatory condition (1, 3).

Among the potential triggers of the autoimmune activation, many infectious agents such as group A streptococcus, *Mycoplasma pneumoniae*, influenza, human herpesvirus 6 (HHV-6), herpes simplex virus (types 1 and 2), parvovirus B19, coxsackievirus, *Borrelia* (Lyme disease), Epstein–Barr virus, cytomegalovirus, and *Candida* have been considered. These human pathogens share neuroinvasive and neurotropic properties, so the CNS can be reached by the blood, or by peripheral or cranial nerves, leading to neuropathological consequences in vulnerable populations (4).

Based on the heterogeneous pathogenesis, treatment of PANS includes different approaches: antibiotics to remove the potential source of neuroinflammation, anti-inflammatory, and immunomodulatory treatments to regulate the immune system, and psychiatric medications to provide symptomatic relief (1).

Given the frequent association between viral/bacterial infections and neuroimmunological disorders, urgent attention should be focused on the coronavirus disease 2019 (COVID-19).

SARS-CoV-2 is an enveloped, positive-sense, single-stranded RNA virus related to many types of infections ranging from asymptomatic or mild flu-like presentation to severe pneumonia and acute respiratory distress syndrome. The occurrence of neurological and psychiatric disorders is increasingly reported in patients with COVID-19, with possible involvement of both central and peripheral nervous systems, leading to the onset of encephalitis, stroke, cerebral vasculitis, headache, seizures, ageusia, anosmia, myalgia, paraesthesia and Guillain–Barre syndrome (4).

In a recent meta-analysis, common neurological post-COVID-19 symptoms have been reported, such as fatigue (37%, 95% CI: 24%−50%), brain fog (32%, 9%−55%), memory issues (27%, 18%−36%), attention disorder (22%, 10%−34%), myalgia (18%, 4%−32%), anosmia (12%, 7%−17%), dysgeusia (11%, 4%−17%), headache (10%, 1%−21%), and several neuropsychiatric conditions including sleep disturbances (31%, 18%−43%), anxiety (23%, 13%−33%), and depression (12%, 7%−21%) (5).

The present study aims (1) to investigate SARS-CoV-2 infection as a risk factor for the pediatric acute-onset neuropsychiatric syndrome (PANS) and (2) to explore the efficacy of steroid treatment on PANS symptoms associated with SARS-CoV-2 infection. The opportunity to screen for SARS-CoV-2 infection in all children and adolescents with acute-onset psychiatric disorders or with a relapse of previous psychiatric symptoms is discussed.

## Materials and methods

This is a naturalistic study based on a clinical database of referred children and adolescents diagnosed as PANS or presenting a clinically significant worsening of previous PANS symptoms after COVID-19 infection, referred to the Child and Adolescent Neuropsychiatry Department at the “A. Cao” Hospital—“G. Brotzu” Hospital Trust, settled in Cagliari (Italy) or to the Child and Adolescent Psychiatry Department of the Scientific Institute “Stella Maris” in Pisa (Italy). Inclusion criteria were: (i) diagnosis of PANS according to the criteria proposed by the 2015 Consensus Conference, (ii) certified COVID-19 positivity, and (iii) average cognitive profile (valued by Wechsler series scales, WISC-IV).

The sample consisted of 10 patients (eight boys and two girls; age range 6–15 years; mean age 9.7 years, SD 2.45), diagnosed with PANS or with worsening of PANS symptoms after COVID-19 infection. All patients had COVID-19 infection between October 2021 and March 2022, certified with a specific test; seven of them have shown mild COVID-19-related symptoms (headache, sore throat, fever, rhinorrhea, and cough), while three of them remained asymptomatic.

Information regarding personal data (i.e., gender and date of birth), COVID-19 data (date of positivity and SARS-CoV-2 symptoms), personal history (i.e., recurrent infections during childhood and a previous diagnosis of PANS), and familiar history (autoimmune and/or psychiatric conditions) have been collected.

All participants were screened for psychiatric disorders using historical information and a structured clinical interview according to DSM-5 criteria, the Schedule for Affective Disorders and Schizophrenia for School-Age Children-Present and Lifetime Version [K-SADS-PL (6)]. Obsessive-compulsive disorder was assessed with Children's Yale-Brown Obsessive-Compulsive Scale (CY-BOCS), considering the score ≥7 as the threshold (7). Tic disorder and Tourette syndrome were assessed with the Yale Global Tic Severity Scale (YGTSS) (8) for the evaluation of tics, considering the score ≥20 as a threshold; and ADHD symptoms were assessed with the Conners' Parent Rating Scale revised short (CPRS-R:S) (9).

Dimensional psychopathology, including both internalizing and externalizing symptoms, was assessed using the Child Behavior Checklist (CBCL) (10), completed by parents.

All PANS symptoms were also specifically investigated, including either obsessions/compulsions, restrictive food intake, or both symptoms, and other symptoms among anxiety, mood disorders, irritability, behavioral/educational regression, sensory integration disorder, motor anomalies, urinary symptoms, sleep disorders, brain fog, heart rate and/or blood pressure alterations, and mydriasis. The Pediatric Acute-onset Neuropsychiatric Syndrome Scale (PANSS) (11) was used for the evaluation of specific PANS symptoms (score ≥20 as the threshold with a possible maximum score of 54).

The level of impairment of global functioning was evaluated by Children's Global Assessment Scale (C-GAS) (12) and the Clinical Global Impression Severity Scale (CGI-S) (13).

Finally, laboratory data have been collected through blood chemistry examination (Table 1), and nasal and pharyngeal swabs.

**Table 1:** Diagnostic procedure.

Routine blood test	Blood counts, sideremia, erythrocyte sedimentation rate (ESR), C-reactive protein (CRP), D-vitamin, free thyroxine (fT4), thyroid-stimulating hormone (TSH), neuron-specific enolase (NSE)
Immunological parameters	Anti-TSH receptor antibodies (AbTR), anti-thyroid peroxidase (AbTPO), anti-thyroglobulin antibodies (AbTG), anti-nuclear antibodies (ANA)
Infectious parameters	Nasal and pharyngeal swab, anti-streptolysin O (ASLO), antibodies (IgM and IgG) anti-herpes simplex virus 1 (HSV-1), Epstein Barr Virus (EBV), *Mycoplasma pneumoniae* and *Chlamydia pneumoniae*

All patients received treatment with corticosteroids (prednisone). For 3 months, monthly oral prednisone pulses of 1(−2) mg/kg daily were administered in one dose on five consecutive days. The baseline assessment was newly administered at the end of the treatment.

This study was conducted in accordance with the Declaration of Helsinki. The Independent Ethical Committee of Cagliari University Hospital approved the study. All parents were fully informed about the study's methods and purposes, and they provided written consent.

### Statistical analyses

Baseline and endpoint results for each patient were compared using a paired *t*-test on continuous variables, with significance set at 0.01 level, two-tailed.

## Results

Seven patients had a previous diagnosis of a neurodevelopmental disorder (autism spectrum disorder, attention-deficit/hyperactivity disorder, Tic disorder, and/or Specific Learning disorder) and one patient had a previous diagnosis of adjustment disorder with anxiety and depressive mood. Two patients had no previous psychiatric diagnosis. Five patients suffered recurrent respiratory airway infections during childhood. Five patients had already received a PANS/PANDAS diagnosis in their history, but they were asymptomatic before the COVID-19 infection onset and they presented an abrupt relapse of PANS symptoms after infection.

A psychiatric family history (mood, behavior, anxiety disorders, and neurodevelopment disorders) was reported in seven patients, and an autoimmune disease family history (mixed connective tissue disease and autoimmune thyroiditis) was reported in three patients.

All patients showed the typical main two PANS symptoms: obsessive-compulsive symptoms in six patients, restrictive food intake in one patient, and both symptoms in three patients. In association, the following symptoms have been reported: anxiety (6/10), mood disorders (7/10), irritability (7/10), behavioral/educational regression (7/10), sensory integration disorder (5/10), tics and motor abnormalities (7/10), urinary symptoms (4/10), sleep disorders (6/10), brain fog (5/10), and mydriasis (1/10).

Laboratory tests were used to explore the role of pathogens other than SARS-CoV-2 in causing PANS symptoms. The nasal swab was negative in all subjects; anti-*M. pneumoniae* antibodies (IgG) were very elevated in one patient; five patients had a slightly elevated anti-streptolysin O titer (ASLO), while the Streptococcus on nasal and pharyngeal swab was negative. One patient showed a high titer of anti-thyroid peroxidase antibodies (AbTPO).

### Treatment

Nine patients were treated with monthly oral prednisone pulses for 3 months; each pulse consisted of 1(−2) mg/kg daily in one dose on five consecutive days. One patient was not treated because his parents did not give a consensus on pharmacological treatment.

All nine patients who received pharmacological treatment were re-assessed 3 months after starting medications.

At the follow-up, among PANS symptoms, sensory integration disorder had the best response to medications (none of the five patients presented this symptom after treatment); also irritability, behavioral/educational regression, and sleep disorders improved after 3 months. Mydriasis was no longer present in the only patient who had it at the baseline. OCD symptoms, tics, and other PANS symptoms consistently diminished even if with lower rates of remission. Anxiety was still present in 5/5 patients, mood disorders in 4/6, motor anomalies in 4/7, urinary symptoms in 3/4, and brain fog in 3/5.

After treatment, we found a clinical improvement in general functioning in all patients. The C-GAS mean score improved from 49.89 ± 9.21 to 75.44 ± 10.81 (difference = 25.56 ± 8.63; *t* = −8.88, df = 8, *p* < 0.001). Similarly, CGI-S improved from 4.00 ± 1.22 to 2.33 ± 0.70 (difference = 1.67 ± 1.00; *t* = 5.00, df = 8, *p* = 0.001).

On the Pediatric Acute-onset Neuropsychiatric Syndrome Scale (PANSS), the mean score decreased from 65.89 ± 24.86 to 27.67 ± 22.84 (difference = 38.22 ± 24.92; *t* = 4.60, df = 8, *p* = 0.002). In four patients, the PANSS total score after treatment resulted under the clinical threshold of the scale (<20).

As far as concern, the evaluation of obsessive-compulsive symptomatology, at PANS onset, five patients showed a CY-BOCS score over the clinical threshold, and after the treatment, one patient remained unchanged, while the other four significantly improved, and three obtained a score under the cut-off. The CY-BOCS score of the five patients improved from 22.6 ± 3.5 to 14.00 ± 6.2 (difference = 8.6 ± 7.0; *t* = 2.74, df = 4, *p* = 0.052).

Five patients showed tics at the onset of the symptoms, as recorded by the Yale Global Tic Severity Scale (YGTSS). After 3 months of treatment, two patients showed complete remission of tic disorder (YGTSS score: 0). The other three patients obtained a significant clinical improvement of tic disorder. The YCTSS of the five patients improved from 64.00 ± 26.23 to 29.00 ± 29.12 (difference = 35.00 ± 17.07; *t* = 4.58, df = 4, *p* = 0.010).

Consistently, all parents reported a global improvement in symptoms after the steroid treatment, as it is witnessed by the score of the questionnaire they completed before and after treatment. ADHD-ODD symptoms, assessed with the Conners' Parent Rating Scale revised short (CPRS-R:S), showed an improvement at the ODD subscale, from 65.22 ± 14.65 to 50.78 ± 16.41 (difference = 14.44 ± 8.79; *t* = 4.93, df = 8, *p* = 0.001). The improvement in the other CPRS subscales (cognition, hyperactivity, and ADHD index) did not reach statistical significance.

Most of the syndromic and DSM oriented subscales improved on the CBCL, but the most significant improvements were found in the items: affective problems (*p* = 0.002), ADHD (*p* = 0.005), social problems (*p* = 0.002), and total problems (*p* = 0.003).

## Discussion

The aim of this article was to explore, in a consecutive sample of children and adolescents, the PANS symptoms onset or relapse after COVID-19 infection and the symptoms response to steroid treatment. Our data confirm that the PANS feature triggered by COVID-19 has symptoms largely overlapping with those reported in PANS triggered by different agents. It includes OCD and/or eating disorders, associated with a heterogeneous mixture of emotional, behavioral, and physical symptoms (14). Our data also suggest that treatment with oral prednisone is associated with a global improvement, in terms of functional impairment and clinical severity and with PANS specific symptoms ameliorating or resolution. Among the psychiatric symptoms, oppositional and affective symptoms seem to be more sensitive to the treatment (Table 2). Our data also offer an ex-adiuvantibus confirmation of the hypothesis that the post-COVID-19 PANS symptoms may be triggered by immune-inflammatory dysregulation in predisposed subjects.

**Table 2:** Pre- and post-treatment scores.

	**PRE-treatment (mean)**	**SD**	**POST-treatment (mean)**	**SD**	***T*-test**	**df**	***p*-value**
C-GAS	49.89	9.21	75.44	10.81	−8.88	8	**0.000**
CGI-S	4	1.22	2.33	0.70	5.00	8	**0.001**
CY-BOCS	22.6	3.5	14	6.2	2.74	4	0.052
PANSS	65.89	24.86	27.67	22.84	4.60	8	**0.002**
YGTSS (five patients)	64	26.23	29	29.12	4.58	4	0.010
**CPRS:**							
Oppositional	65.22	14.65	50.78	16.41	4.93	8	**0.001**
Cognitive problems	64.11	21.72	61.56	16.33	0.60	8	0.562
Hyperactivity	70.11	22.42	62.33	17.85	2.21	8	0.058
ADHD index	69.89	23.47	61.56	14.09	1.52	8	0.168
**CBCL DSM-5 scales**							
Affective problems	66.00	8.66	55.11	7.20	4.50	8	**0.002**
Anxiety problems	65.33	10.01	58.22	12.08	3.71	8	0.006
Somatic problems	58.44	12.93	54.33	7.28	1.49	8	0.176
ADHD	65.44	11.62	58.33	9.31	3.89	8	**0.005**
Oppositional-defiant problems	61.11	10.79	54.56	8.78	2.37	8	0.045
Conduct problems	58.78	7.73	52.00	3.24	2.46	8	0.039
**CBCL syndrome scales**							
Anxious-depressed	61.22	12.26	56.67	12.53	2.88	8	0.021
Withdrawn-depressed	59.00	8.75	54.56	7.43	2.92	8	0.019
Somatic complaints	62.67	11.08	55.78	7.38	2.93	8	0.019
Social problems	59.56	7.78	56.22	8.53	2.50	8	0.037
Thought problems	66.33	7.56	55.89	8.75	4.56	8	**0.002**
Attention problems	68.22	15.13	57.11	7.29	3.20	8	0.013
Rule breaking behavior	58.56	9.71	54.11	7.32	1.34	8	0.218
Aggressive behavior	61.67	10.75	54.89	7.49	2.87	9	0.021
**CBCL**							
Internalizing problems	61.56	10.60	51.67	13.25	3.63	8	0.007
Externalizing problems	58.67	13.14	49.00	12.18	2.47	8	0.025
Total problems	62.89	10.46	51.00	13.53	4.09	8	**0.003**

CGI-S, Clinical Global Impression–Severity score; C-GAS, Children Global Assessment Scale; CY-BOCS, Children Yale-Brown Obsessive-Compulsive Scale; YGTSS, Yale Global Tic Severity Scale; PANSS, Pediatric Acute-onset Neuropsychiatric Syndrome Scale; CPRS, Conners' Parent Rating Scale; CBCL, Child Behavior Checklist.

Statistical significance at p < 0.01.

Bold values mean statistical significance at the p < 0.05 level.

Since the PANS onset can occur as a post-/peri-infectious autoimmune/inflammatory condition, it is intuitive that among the possible agents triggering the PANS symptomatology, COVID-19 must be included. It can represent a trigger for developing PANS symptoms, as already suggested by other research groups (15, 16). Regarding etiopathogenesis, a recent study (17) suggests a possible genetic role of some “PANS gene” as *PPM1D, CHK2, RAG1*, and *PLCG2*, highly expressed in peripheral immune responses and microglia and the enteric nervous system and the choroid plexus. Genetic variation in PANS candidate genes may function by disrupting peripheral and central immune functions, neurotransmission, and/or the blood-CSF/brain barriers following stressors such as infection. According to the authors, the expression of several of these genes was also markedly altered in a sample of patients with SARS-CoV-2 infection, suggesting a shared possible liability of some individuals in developing both PANS and SARS-CoV-2 symptoms.

A recent report (18) highlighted that pediatric patients with COVID-19 and prominent sub-acute neuropsychiatric symptoms, ranging from severe anxiety to delusional psychosis, may have anti-SARS-CoV-2 and antineural antibodies in their CSF and may respond to immunotherapy. Nevertheless, the plausible pathogenic mechanism is not based on the SARS-CoV-2 infiltration into the CNS or antibody production, but on the activation of neuroinflammatory response due to the peripheral cytokines that reach the cerebral parenchyma due to a lack of BBB integrity. Consequently, the transmigration of peripheral immune cells into the CNS and the microglia activation cause the alteration of neurotransmission (19).

The small sample size, as well as the relatively brief follow-up, from baseline to the endpoint, strongly limits our conclusion. Furthermore, as PANS presentation can be episodic, we could not exclude that the improvement may be due to a spontaneous positive evolution of the disorders after the acute phase. Noteworthy, the acute onset of typical PANS symptoms after SARS-CoV-2 infection allows us to infer that early steroid treatment may have had some effect, according to the previous data from large PANS sample sizes suggesting that steroids, given for the initial PANS flare, significantly shorten the acute episode when compared to no treatment condition (20).

In conclusion, our study suggests that COVID-19 infection should be explored in children and adolescents with acute-onset neuropsychiatric disorders. Conversely, in children and adolescents with COVID-19, a specific neuropsychiatric follow-up should be routinely included in the clinical management of these young patients. Finally, in patients with more severe and acute symptoms, corticosteroid treatment in the acute phase may be beneficial and well tolerated.

## Data availability statement

The original contributions presented in the study are included in the article/supplementary material, further inquiries can be directed to the corresponding author.

## Ethics statement

This study was conducted in accordance with the Declaration of Helsinki. The Independent Ethical Committee of Cagliari University Hospital approved the study. All the parents were given a full explanation of the study methods and purposes and they gave their written consent.

## Author contributions

Conceptualization and methodology: SB, AG, and GM. Formal analysis: GP, GM, and AS. Investigation: SB, AG, GM, AS, GP, LF, FN, and MR. Data curation: SB, AS, and GM. Writing—original draft preparation: AS, GP, LF, FN, and MR. Writing—review and editing and supervision: AG and GM. Funding acquisition: AG. All authors have read and agreed to the published version of the manuscript.
