# Corrigendum: Steroid treatment response to post SARS-CoV-2 PANS symptoms: Case series

**DOI:** 10.3389/fneur.2023.1178013

**Published:** 2023-03-27

**Authors:** Stefano Berloffa, Andrea Salvati, Gloria Pantalone, Ludovica Falcioni, Micaela M. Rizzi, Francesca Naldini, Gabriele Masi, Antonella Gagliano

**Affiliations:** ^1^IRCCS Stella Maris, Scientific Institute of Child Neurology and Psychiatry, Pisa, Italy; ^2^Department of Clinical and Experimental Medicine, University of Pisa, Pisa, Italy; ^3^Child and Adolescent Neuropsychiatry Unit, Department of Biomedical Science, University of Cagliari & “A. Cao” Pediatric Hospital, Brotzu Hospital Trust, Cagliari, Italy; ^4^Neurology Unit, Department of Neurology of Health Science, Magna Graecia University of Catanzaro, Catanzaro, Italy

**Keywords:** SARS-CoV-2, TIC, pediatric acute-onset neuropsychiatric syndrome (PANS), steroid, neuropsychiatric disorder

In the published article, the Funding statement was mistakenly omitted. The correct Funding statement appears below.

This work has been partially supported by a grant from the IRCCS Fondazione Stella Maris (Ricerca Corrente, and the “5 × 1000” voluntary contributions, Italian Ministry of Health).

The authors apologize for this error and state that this does not change the scientific conclusions of the article in any way. The original article has been updated.

